# CBCT and CAD-CAM technology to design a minimally invasive maxillary expander

**DOI:** 10.1186/s12903-020-01292-3

**Published:** 2020-11-04

**Authors:** Diego Sánchez-Riofrío, María J. Viñas, Josep M. Ustrell-Torrent

**Affiliations:** 1 Universitat de Barcelona, Feixa Llarga s/n. Pavelló de Govern 2ª pl. Office 2.7, L’Hospitalet de Llobregat, 08907 Barcelona, Spain; 2 Universidad Espíritu Santo, Samborondón Campus, Km. 2.5 vía La Puntilla, Samborondón, Ecuador; 3 Faculty of Dentistry, Universidad Complutense de Madrid, Ciudad Universitaria, Plaza Ramón y Cajal S/N, Madrid, Spain; 4 Director Master of Orthodontics, Dental School, Universitat de Barcelona, Feixa Llarga s/n. Pavelló de Govern 2ª pl. Office 2.7, L’Hospitalet de Llobregat, 08907 Barcelona, Spain

**Keywords:** CAD/CAM, Bone-borne, RME, Maxillary expander, CBCT, Digital models, 3D printed, Surgical guide, Miniscrews

## Abstract

**Background:**

A large number of articles in recent years studying the effects of non-surgically assisted tooth- versus bone-borne maxillary expanders in growing patients have found no significant differences in mid-palatal suture disjunction or even dentoalveolar changes. This suggests the need for new criteria and better use of current technology to make more effective devices and enhance the benefits of conventional treatments. This article describes a titanium grade V computer-aided design/computer-aided manufacturing (CAD/CAM) maxillary expander supported by two miniscrews, along with a 3D printed surgical guide.

**Methods:**

The first step was to obtain a digitized model of the patient’s upper maxilla. To simplify the process and ensure the placement of the device in a high-quality bone area, the patients’ digital dental cast was superimposed with a cone beam computed tomography (CBCT) scan. Improved resistance to expansion forces was secured through the use of 2 mm-wide miniscrews, long enough for bicortical anchorage. Placement site and direction were assessed individually in order to achieve primary stability. We chose a site between the second premolars and first molars, while the inclination followed the natural contour of the palate vault. A 3D-printed, polyamide surgical guide was designed to ensure the correct placement of the device with a manual straight driver.

**Results:**

Favorable clinical results were presented with 3D images. We confirmed a mid-palatal suture parallel separation of 3.63 mm, along with a higher palatal volume, as well as increased intercanine and intermolar distance. Segmentation of the facial soft tissue showed an expansion of nasal airways and changes in nasal morphology.

**Conclusions:**

Digital models, CBCT and CAD/CAM technology, are essential to accomplish the goals proposed in this article. Further studies are necessary to establish safer miniscrew placement sites and insertion angles so as to achieve greater in-treatment stability. Both the clinician and the patient can benefit from the use of current technology, creating new devices and updating traditional orthodontic procedures.

## Background

For more than a century, tooth-borne devices have been used to achieve palatal disjunction, increase intercanine width, correct maxillary constriction and posterior crossbite [1]. Benefits from this treatment include solving positional and functional mandibular deviations, facial asymmetries and altered dentofacial aesthetics [2].

Nevertheless, studies conducted on adolescents and young adult patients show that these devices decrease the amount of force applied in the maxilla, causing a major dental impact along with other undesirable periodontal side effects [3, 4]. It has been suggested that using alternative bone-borne expanders may cause direct skeletal changes in these patients, with a lower dental expansion component [5, 6].

After many years studying the effects of tooth- versus bone-borne devices, results are still controversial. In growing patients, separation of the mid-palatal suture has proven effective with both devices, and neither group has shown statistically significant dentoalveolar bone changes [7–9]. These results have brought to light the need for other criteria to justify the use of bone-borne maxillary expanders in early adolescents, such as: the size of the device for better comfort, fewer anatomical structures supporting the device (teeth, palate, number of miniscrews, etc.) and cost-effectiveness regarding dentist chair time, among others.

In light of these requirements, we describe a Computer-Aided Design/Computer-Aided Manufacturing (CAD/CAM) maxillary expander supported by two miniscrews. This technology allowed us to customize the device, adapting it to the palate vault of the patient and reducing its size for increased effectiveness [10]. Aligning a Cone Beam Computed Tomography (CBCT) with a digitized model of the upper maxilla allowed us to determine the exact position of the miniscrews, ensuring high-quality bone support.

We used a 3D printed in polyamide surgical guide to ensure correct expander placement. Different authors have described the advantages of using surgical guides when placing miniscrews, concluding that the use of CBCT and digital models improved the final outcome [11, 12].

Even though in the palate there is no risk of injuring the roots of adjacent teeth, CBCT allows measuring palatal bone thickness while also facilitating the correct direction of miniscrew insertion [12, 13], both important aspects to achieve primary stability of the device.

The aim of this study was to report all stages of the manufacturing process of a CAD/CAM midpalatal disjunction device and a 3D-printed surgical guide. We also conducted a 3D assessment of the expansor’s effects by using CBCT images and superimposed digital models.

## Methods

This study was approved by the Hospital Odontològic Universitat de Barcelona Ethics Committee CEIC, No. 2017/34. The first step was to obtain a digitized model of the patient’s upper maxilla. We took a conventional alginate impression and later digitized the model cast. Then, we used a 3Shape D2000 tool to scan the dental casts and the 3Shape Dental System v.2017 software to create the digital models.

Before initiating the orthodontic treatment, we took a CBCT (Planmeca series No. TFMP1015B) with a volume of 150 × 100x80 mm, which covered two thirds of the maxillofacial skeleton. Another similar CBCT was taken to compare the results immediately after the palatal expansion. Both records had a voxel size of 400 μm to achieve high-quality 3D image composition, and were developed using the Mimics Medical v20.0 software. Digital casts and CBCT records were superimposed with the 3-Matic Medical v12.0 tools.

**Fig. 1:**
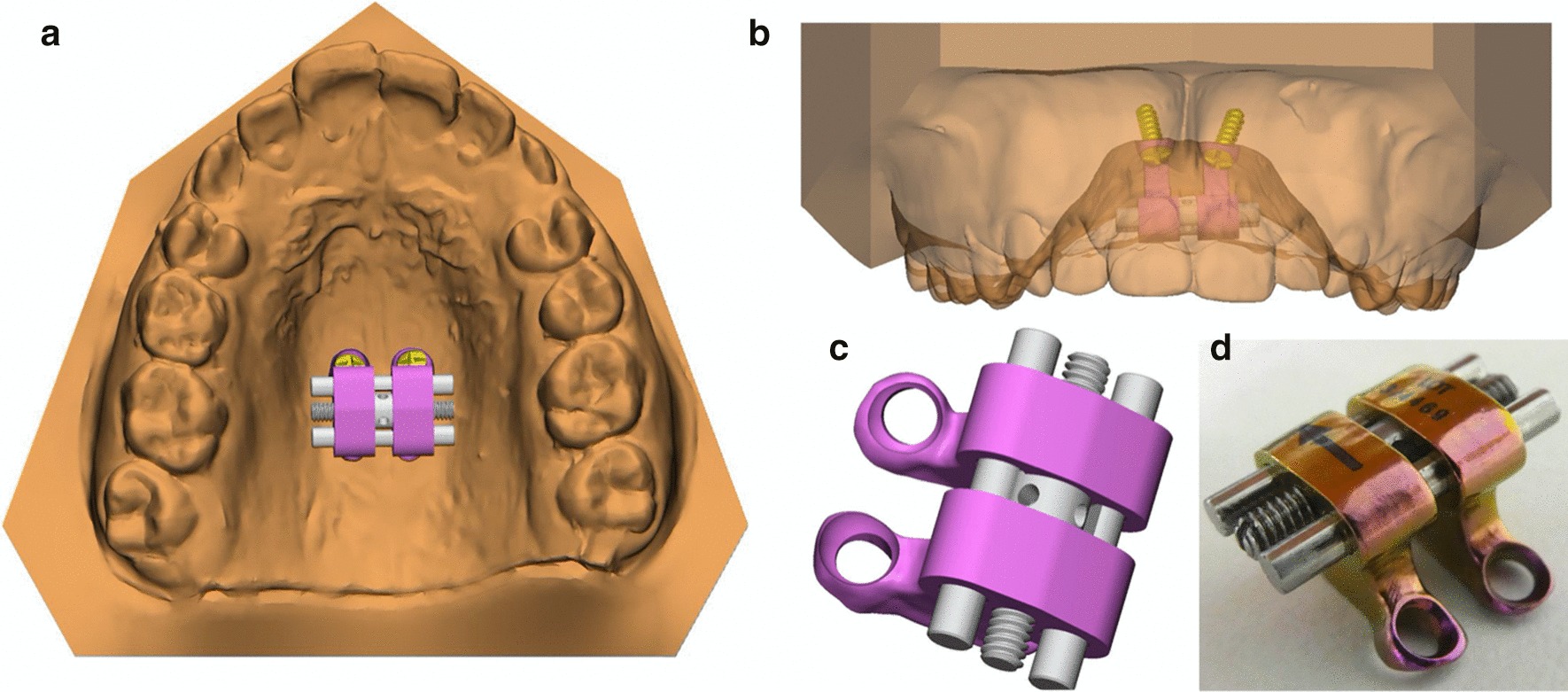
**a** Occlusal view of the maxillary expander in the digital model of the upper maxilla. **b** Frontal view of the digital model exposing the device and the two miniscrews. **c** Computer aided design. **d** Final product after manufacture

For the sake of comfort, we used a very small maxillary expander with only two miniscrews for bone-borne anchorage. The shape of the maxillary arch, whose bottom was deep and narrow, required separating the expander from the palate vault. The device therefore consisted of a conventional Hyrax-type screw (designed by Forestadent, Pforzheim, Germany) supported by two pillars or “legs” with two anterior perforations for miniscrew placement, with a total size of 11 × 10.3 × 9.7 mm Fig. 1.

We did not suggest using molar bands or an acrylic pad for additional support. The main challenge was to obtain primary stability of the device with only two miniscrews. To this end, we considered two aspects: the characteristics of the temporary anchorage devices (TADs) and location site.

We decided their characteristics based on several studies. Walter et al. [14], determined that the use of 1.5 mm diameter titanium mini-implants may undergo major deformations with a risk of breakage; we therefore suggested the use of TADs with a diameter > 2.0 mm for bone-borne maxillary expanders. Lee et al. [15] concluded that bicortical mini-implant anchorage is recommended to avoid deformation and fracture. Since length should be proportional to the placement site, we considered the thickness of the cortical bone in the premolar area with a mean value of 1.18 mm, in line with the findings of Johari et al. [16]. Thus, we proposed a length of 8 mm.

**Fig. 2:**
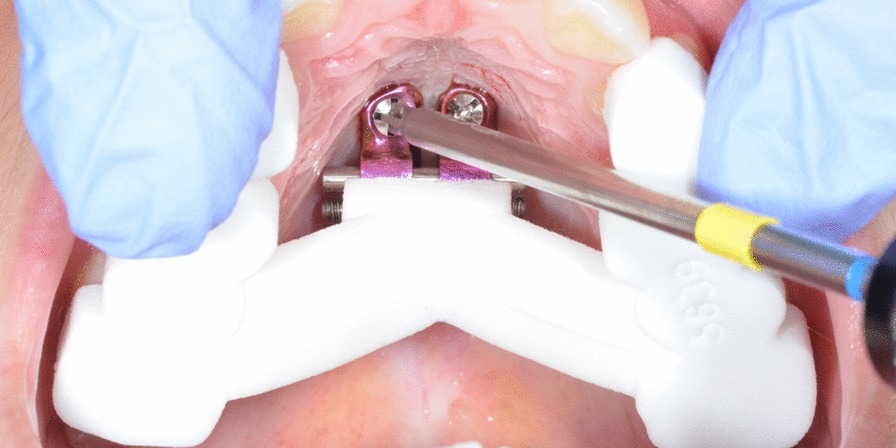
Placement of the maxillary expander and the miniscrews with the 3D printed surgical guide

Miniscrews were inserted with approximately 30º posterior and 20º lateral inclinations, following the natural contour of the palate vault [17]. This allowed us to maximize the amount of bone surface in contact with the miniscrews and to simplify the insertion procedure of the miniscrews using a manual straight driver Fig. 2.

### Production process

We considered two options for production: 3D printing or 5 axis milling. 3D printing was not deemed appropriate since the device included smaller parts that needed further assembly, making us decant for the milling machine instead (HSC 20 linear from DMG MORI). The maxillary expander was made with grade V titanium (Ti6AI4V metal composition). Its inherent properties—e.g., biocompatibility, rigidity, lightness and resistance to the intense forces of palatal disjunction—allowed us to keep the device small while ensuring that it would not collapse during the treatment. We required a completely polished surface with a quality of Ra < 0.0025 μin to ensure comfort and to achieve the best contour and fitting accuracy. For the milling procedure, we used a titanium-aluminum-vanadium based dental alloy KERA® TI 5 disc (Eisenbacher Dentalwaren ED GmbH), a material that is normally used for dental purposes in crowns, bridges and implant-retained suprastructures.

The perforations in the anterior part of the expander were made to fit two 2.0 × 8 mm diameter miniscrews (Jeil, ref 20AT-008). They were designed to provide the correct direction by working as a screw cap system, sealed with the miniscrew heads at the end of the insertion process.

We designed a CAD/CAM surgical guide using the same 3-Matic software to facilitate expander placement and ensure correct miniscrew positioning. It surrounded the occlusal area of both premolars and molars bilaterally while safely securing the maxillary expander at the center, allowing the clinician to operate easily.

The surgical guide was manufactured with laser sintering 3D printing technology (Formiga printer P110 from EOS). We used polyamide (PA2200), a multipurpose material with a balanced property profile that is very strong and stiff, has good chemical resistance, high selectivity, detailed resolution and is biocompatible. This technique uses a fiber laser to melt and fuse fine plastic powder. We built the 3D object by layers based on the computer-aided design. Each layer was 0.1 mm thick, so that a sphere with a 1 cm diameter had 100 layers, thus providing maximum printing precision and fit—especially in the molar cusp and groove areas. The final size of the surgical guide was 34.7 × 44.5 × 14.5 mm.

### Clinical example

The patient, a 13-year-old Caucasian-Spanish female, attended the Orthodontic Department of the Hospital Odontològic Universitat de Barcelona in Catalunya, Spain. Her parents were mainly concerned about her loud snoring, caused by poor nasal breathing, and difficulties in chewing due to a posterior crossbite and an augmented overjet.

The cephalometric analysis revealed a skeletal Class II with a high A point convexity and a short mandibular length. There was a moderate Class II molar and canine relationship, with an augmented overjet of 7 mm, maxillary-mandibular arch length discrepancy and vestibularized incisors. V-shaped maxillary and U-shaped mandibular arch forms were asynchronous. No temporomandibular joint disorder symptoms and signs could be observed on radiographic and clinical evaluations.

The treatment objectives were to correct the posterior crossbite and expand the maxilla, preparing it for future mandibular advancement. We decanted for rapid maxillary expansion (RME) as the most suitable choice, to even the arch length discrepancy, change the V-shaped maxillary arch and improve nasal breathing [18, 19].

The treatment started after the patient’s parents decided to participate in this study and signed a written informed consent according to ethical principles.

To ensure an effective RME, we started by correcting the negative torque of the posterior lower teeth. Using fixed braces in the lower arch and posterior build-ups to decompensate, the real posterior crossbite was obtained as a reference of how much maxillary expansion was truly needed. After placing a lower rectangular 0.019 × 0.025-inch stainless steel arch, we began production of the CAD/CAM device following the steps described in the previous section.

Once the customized palatal expander, the surgical guide and the two miniscrews were ready, we were able to place the device in just one visit.

First, we made sure that the surgical guide fitted perfectly with the device and checked that they were both stable in the patient’s mouth. To avoid patient discomfort, we applied topical gel and local anesthesia (articaine 4%—epinephrine 0,5%) in the surrounding palate area before inserting the miniscrews.

**Fig. 3:**
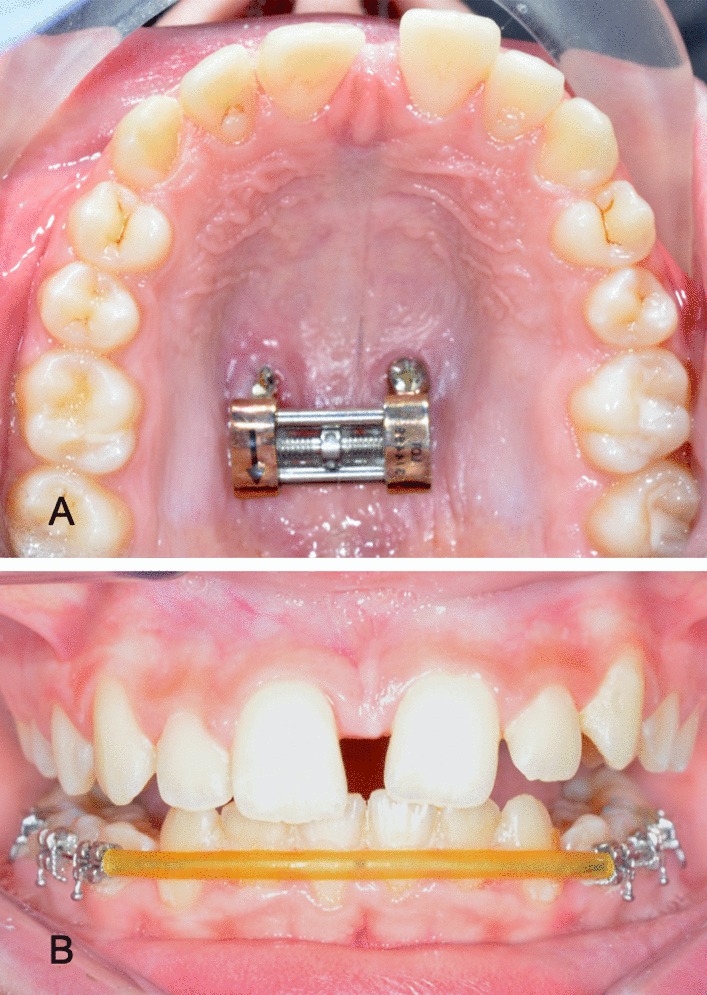
**a** Occlusal view at the end of the activation phase. **b** Frontal view asking the patient to advance the mandible to confirm complete correction of posterior crossbite

We used a conventional RME protocol for activation: a 90º turn of the central screw twice a day for a daily expansion of 0.5 mm over two weeks, to obtain a total expansion of 7 mm. At the end of this process, we asked the patient to advance the mandible, simulating the final result after the use of a functional device for Class II correction, so as to verify that palatal disjunction was adequate. Figure 3.

## Results

**Fig. 4:**
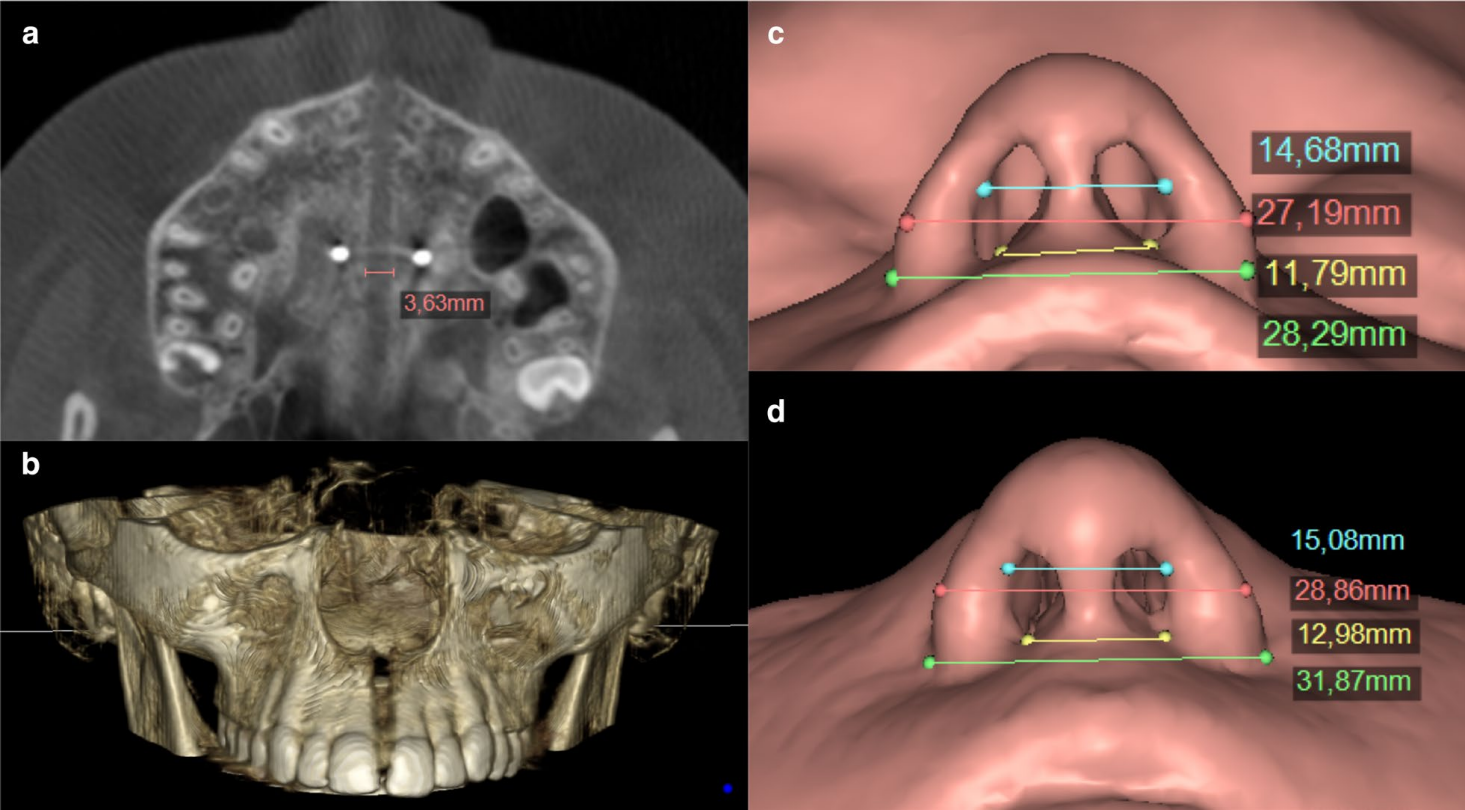
**a** CBCT occlusal view showing parallel separation of the mid-palatal suture. **b** 3D composition of the upper maxilla after RME (frontal view). **c** Landmarks and measurements showing changes of the nose and nostrils before RME. **d** Nasal morphology changes after RME.

After the activation period ended, we took a second CBCT for control. Immediate changes after palatal disjunction were analyzed by measuring the opening of the midpalatal suture on the transversal plane (Nemoscan v18. Nemotec. Madrid, Spain), revealing a parallel separation of 3.63 mm. Figure 4A, 4B. This vertical pattern is similar to findings described in literature and confirmed a major skeletal effect of the expansion [4, 5, 20]. The patient and her parents reported improved nasal breathing and no more snoring. Using data from Kim et al. study on nasal airways [19], we also performed a segmentation of facial soft tissue. Nasal morphology change analysis results were positive as shown in Table 1. Figure 4C, 4D.

**Table 1:** Pre- and post-activation changes in nasal morphology

	Initial CBCT	Final CBCT
Alar (mm)	28.29	31.87
Alar curvature (mm)	27.19	28.86
Nostril superior (mm)	14.68	15.08
Nostril inferior (mm)	11.79	12.98

**Fig. 5:**
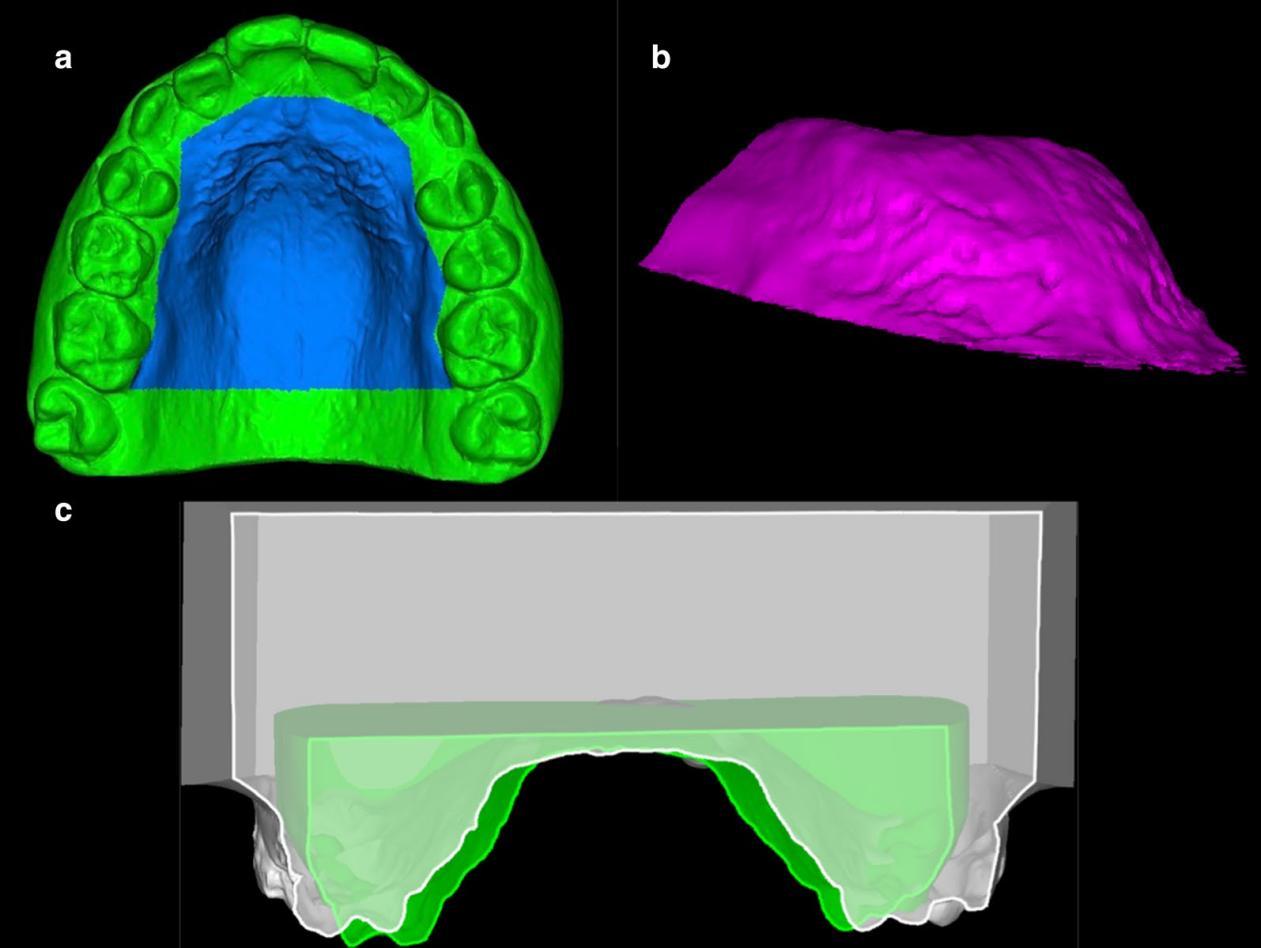
**a** Initial model with delineated area for evaluation of changes in the palate. **b** Separated volume of the palatal area of the final digital cast. **c** Superposition of both records showing differences in the palate vault, six months after RME activation protocol

The retention protocol lasted for 6 months. After removal of the device, we took and digitized a second dental cast. Superimpositions of both digital models were performed in two stages using the Nemocast v18 software (Nemotec. Madrid, Spain) [21]. We first selected similar landmarks on the palatal rugae of the source and the reference meshes, and performed preliminary calculations. 5 points per mesh were marked. The software then calculated the rigid transformation and minimized the matching error, which was represented numerically to check for accuracy. The second phase used a best-fit algorithm to superimpose the models. The software found the final rigid transformation that best adjusted the surface on the reference mesh. Superimposition of the digital models showed stability of the palatal disjunction after 6 months. Figure 5.

Intercanine and intermolar distance were also analyzed. Intercanine distance was measured using a central point in the incisal margin vertices of both canines and a straight line joining both marks. Intermolar distance on the first molars was measured using similar points in the mesiovestibular cusp vertices and a straight line joining both marks. Table 2 shows the increase in these values along with palatal volume and area.

**Table 2:** Comparison of both digital models pre- and post RME treatment

	Initial model	Treatment model
Intercanine distance (mm)	31.99	36
Intermolar distance (mm)	47.63	53.15
Palatal area (mm^2^)	1.248,94	3.464,79
Palatal volume (cc^3^)	5.849	6.718,30

## Discussion

Using current technology to simplify traditional procedures in orthodontics can be challenging, especially with demanding objectives such as those proposed in this article.

The main objective was to achieve primary stability with two miniscrews. The anterior part of the palate vault is considered the best area for mini-implant placement, even though there is massive inconsistency and high individual variability of bone thickness according to age, gender and location sites [13, 17].

For palatal disjunction, a posterior position of the device is convenient to obtain a parallel separation of the mid-palatal suture. Since some studies have suggested a posterior inclination of the miniscrews [10, 16], we decided on a placement site between the second premolars and a lateral miniscrew insertion with a slight posterior tilt, following the natural contour of the palate vault. Aligning the digital models with the CBCT helped us to more accurately assess bone quality before deciding on the final placement and direction of the miniscrews.

If no additional TADs, palate pads or molar bands are added to the expander, an individual evaluation of bone quality with CBCT is recommended in every patient before deciding the placement site.

During the activation period, we detected some minor device instability. We corrected this by adjusting the miniscrews about half a turn each at the end of the second week. We observed mild inflammation of the surrounding soft tissue due to contact with the miniscrews and the expander. Similar outcomes were found in the literature [22]. Treatment with chlorhexidine gel 0.2% for 1 week was indicated. An evaluation of soft tissue—especially thickness and mobility—is also recommended before selecting the miniscrew insertion site.

Current bone-borne maxillary expanders use other structures to guarantee primary stability: more miniscrews, molar bands attached with metal arms, palatine pads, among others. They have undesirable side effects that compromise patient comfort and complicate clinical procedures. Using these digital tools to perform an individual analysis of hard and soft tissue allows creating a bespoke device that can simplify the entire treatment.

Using a 3D-printed surgical guide was another essential tool to achieve our goal. According to the literature, most bone-borne maxillary expanders required at least two visits to determine the placement site, create records with the miniscrews placed in the palate and then obtain the final design. CAD/CAM technology allowed us to design and plan everything in a single visit, while the 3D printed surgical guide allowed us to place the expander and the miniscrews in the same day, confirming their precise position according to digital planning. The accuracy of this technique is discussed by Cassetta et al. [23], who conclude that it lacks a “learning curve” effect, thus making it a simple procedure with a very low risk of failure.

Clinical results obtained in this study need to be ascertained with a larger sample in order to establish the palatal expander efficiency; however, conclusions are directed towards methods and materials used and how they can be managed to improve current clinical procedures.

## Conclusions

1. Digital models, CBCT and CAD/CAM technology, are essential to accomplish an effective palatal disjunction device, which can be easily placed in one visit.
2. Due to individual variability, a patient CBCT superimposed with a digital model are vital to determine the best miniscrew placement site in each case.
3. There must be an assessment not only bone and cortical structures, but also of soft-tissue thickness and mobility.
4. Further studies are required to establish safer miniscrew placement sites and insertion angles to achieve in-treatment stability.
5. Both the clinician and the patient can benefit from the use of current technology to create new devices and update traditional orthodontic procedures.

## Data Availability

The datasets used and/or analyzed during the current study are available from the corresponding author on reasonable request.

## Abbreviations

CAD/CAM: Computer-aided design/computer-aided manufacturing
CBCT: Cone beam computer tomography
TADs: Temporary anchorage devices
RME: Rapid maxillary expansion
